# Clinical Application of Inflammatory Vital Pulp Therapy in Immature Permanent Teeth: A Retrospective Case Series

**DOI:** 10.1155/crid/5831702

**Published:** 2026-09-09

**Authors:** Yibing Wang, Wenqi Zheng, Xiangzi Zhang

**Affiliations:** ^1^ Department of Stomatology, The Affiliated Hospital of Yanbian University (Yanbian Hospital), Yanji, Jilin, China; ^2^ Department of Stomatology, College of Medicine, Yanbian University, Yanji, Jilin, China, ybu.edu.cn

**Keywords:** double antibiotic paste, immature permanent teeth, inflammatory vital pulp therapy, root development, stem cells from the apical papilla

## Abstract

**Background:**

Management of immature permanent teeth with pulpal or periapical pathology remains challenging due to incomplete root development and open apices. Conventional treatments, such as calcium hydroxide apexification, require multiple appointments over extended periods and may weaken dentinal walls, whereas apical barrier techniques using mineral trioxide aggregate (MTA) provide apical closure but do not promote continued root development. Inflammatory vital pulp therapy (IVPT) has emerged as a biologically based approach that preserves stem cells from the apical papilla (SCAPs) to promote continued root maturation.

**Case Presentation:**

This retrospective case series included four male patients (aged 8–12 years) with immature permanent teeth diagnosed with pulpal or periapical disease. All cases were treated using a standardized IVPT protocol involving canal disinfection with double antibiotic paste while preserving apical vital tissue. Clinical and radiographic outcomes were evaluated over a mean follow‐up period of 32.5 months (range: 22–48 months).

**Results:**

All cases achieved resolution of clinical symptoms and periapical healing. Three cases demonstrated complete apical closure and continued root development, whereas one case showed partial success with periapical healing but incomplete apical closure and mild tooth discoloration.

**Conclusions:**

IVPT may represent a promising treatment option for immature permanent teeth with pulpal or periapical pathology. By preserving viable apical tissues and controlling infection, it supports physiological root maturation. Further studies with larger sample sizes and longer follow‐up are warranted.

## 1. Introduction

Immature permanent teeth are characterized by incomplete root development, open apices, and thin dentinal walls, which make endodontic treatment particularly challenging. The absence of an apical constriction compromises effective root canal obturation and increases the risk of material extrusion, potentially impairing periapical healing and root development [1].

Current treatment strategies for immature permanent teeth with pulpal or periapical pathology include apexification, apical barrier techniques, and regenerative endodontic treatment (RET) [2]. Calcium hydroxide apexification requires multiple visits over an extended period (6–24 months) and may weaken dentinal walls without promoting further root development [3]. Apical barrier techniques using MTA allow single‐visit treatment but do not facilitate continued root maturation [4]. RET aims to regenerate pulp‐like tissue and promote root development; however, it involves complex procedures, such as induction of intracanal bleeding, and its outcomes remain variable [5].

Inflammatory vital pulp therapy (IVPT) has emerged as an innovative treatment strategy based on contemporary understanding of dental stem cell biology [6]. The fundamental principle of IVPT is the preservation of stem cells from the apical papilla (SCAPs), which may remain viable even in the presence of pulp necrosis and infection. The apical papilla possesses unique biological characteristics that support its survival, including collateral blood supply from periodontal tissues, lower metabolic demands, and proximity to highly vascularized dental follicles. These features enable SCAPs to survive in adverse conditions and contribute to continued root development once infection is controlled [7].

Previous studies have demonstrated that root maturation can occur despite persistent periapical inflammation, suggesting that SCAPs retain functional potential even in compromised environments.

Therefore, this retrospective case series is aimed at evaluating the clinical and radiographic outcomes of IVPT in immature permanent teeth, focusing on its effectiveness in infection control and promotion of root development.

## 2. Case Presentation and Methods

### 2.1. Study Design and Case Selection

This retrospective case series was conducted at the Department of Stomatology, Affiliated Hospital of Yanbian University, between October 2020 and December 2022. The study was approved by the Science and Technology Ethics Committee of the Affiliated Hospital of Yanbian University (Approval No.: 20260261) and conducted in accordance with the Declaration of Helsinki. Written informed consent for treatment and publication was obtained from the parents or legal guardians of all patients. All patient information was anonymized prior to analysis.

The inclusion criteria were as follows: (1) patients aged 6–14 years with immature permanent teeth; (2) open apices corresponding to Nolla developmental Stage 8 or 9, confirmed by periapical radiographs; (3) clinical and radiographic diagnosis of irreversible pulpitis or apical periodontitis necessitating endodontic intervention; (4) treatment performed according to a standardized IVPT protocol (see Section 2.2); and (5) availability of complete clinical and radiographic follow‐up data for a minimum of 22 months.

The exclusion criteria were the following: (1) teeth with complete root development (Nolla Stage 10 or apical closure confirmed radiographically); (2) patients with systemic diseases that could affect healing or immune response (e.g., uncontrolled diabetes and immunodeficiency disorders); (3) teeth with root fractures, extensive internal or external root resorption, or nonrestorable coronal structure; and (4) cases with incomplete or missing follow‐up records. After applying these criteria, four cases were eligible for inclusion in this case series.

### 2.2. Treatment Protocol

All treatments were performed by a single experienced operator under local anesthesia (2% lidocaine with 1:100,000 epinephrine) with rubber dam isolation. The standardized IVPT protocol comprised the following steps:

1. Access and disinfection: After conservative access cavity preparation, necrotic pulp tissue and debris were carefully removed from the coronal and middle thirds of the canal without instrumentation beyond the estimated working length, to avoid damaging residual vital tissue in the apical region. The presence of residual vital tissue in the apical region was confirmed clinically by bleeding characteristics during access preparation. The canal was gently irrigated with 1% sodium hypochlorite (NaOCl) followed by sterile saline. A concentration of 1% NaOCl was selected to balance antimicrobial efficacy with biocompatibility, as higher concentrations have been reported to be cytotoxic to SCAPs and may compromise the survival of residual vital tissue [8, 9].
2. Intracanal medication: A double antibiotic paste (DAP) was prepared by mixing metronidazole and ciprofloxacin powders in a 1:1 ratio by weight, followed by addition of sterile saline until a creamy consistency was obtained. The amount of saline was adjusted clinically to achieve appropriate handling properties. The paste was gently placed over the remaining vital tissue in the apical region using a Lentulo spiral, with care taken to avoid excessive pressure or extrusion beyond the apex.
3. Temporary restoration and recall: The access cavity was temporarily sealed with zinc oxide–eugenol cement. Patients were recalled after 2–3 months. If clinical symptoms had resolved and the temporary restoration remained intact, the intracanal medication was removed and the tooth was semipermanently restored with glass ionomer cement (GIC). GIC was selected as the restorative material because of its chemical adhesion to tooth structure, fluoride release for caries prevention, and suitability as a semipermanent restoration in growing patients with immature teeth, where definitive composite restorations may be considered after complete root maturation [10]. If symptoms persisted, the intracanal medication was replaced once, and the patient was recalled after an additional 1–2 months before definitive restoration.

### 2.3. Follow‐Up and Outcome Assessment

Patients were followed up at 1, 3, 6, and 12 months postoperatively, and every 6–12 months thereafter. Clinical examinations included percussion, palpation, mobility assessment, and pulp vitality testing.

Periapical radiographs were obtained using a standardized digital radiography technique with a paralleling method and positioning device to ensure reproducibility. Root development was assessed using the Nolla staging system.

Treatment outcomes were categorized according to criteria adapted from previously published studies on regenerative endodontic procedures [11] as follows:

1. Success: complete resolution of clinical symptoms, healing of periapical lesions (disappearance of radiolucency), continued root development, and apical closure.
2. Partial success: absence of clinical symptoms with periapical healing, but incomplete apical closure or occurrence of complications such as tooth discoloration.
3. Failure: persistence of clinical symptoms or enlargement of periapical lesions, requiring alternative treatment (e.g., apexification, apical barrier technique, or extraction).

### 2.4. Case Reports

#### 2.4.1. Case 1 (Figure 1)

**Figure 1:**
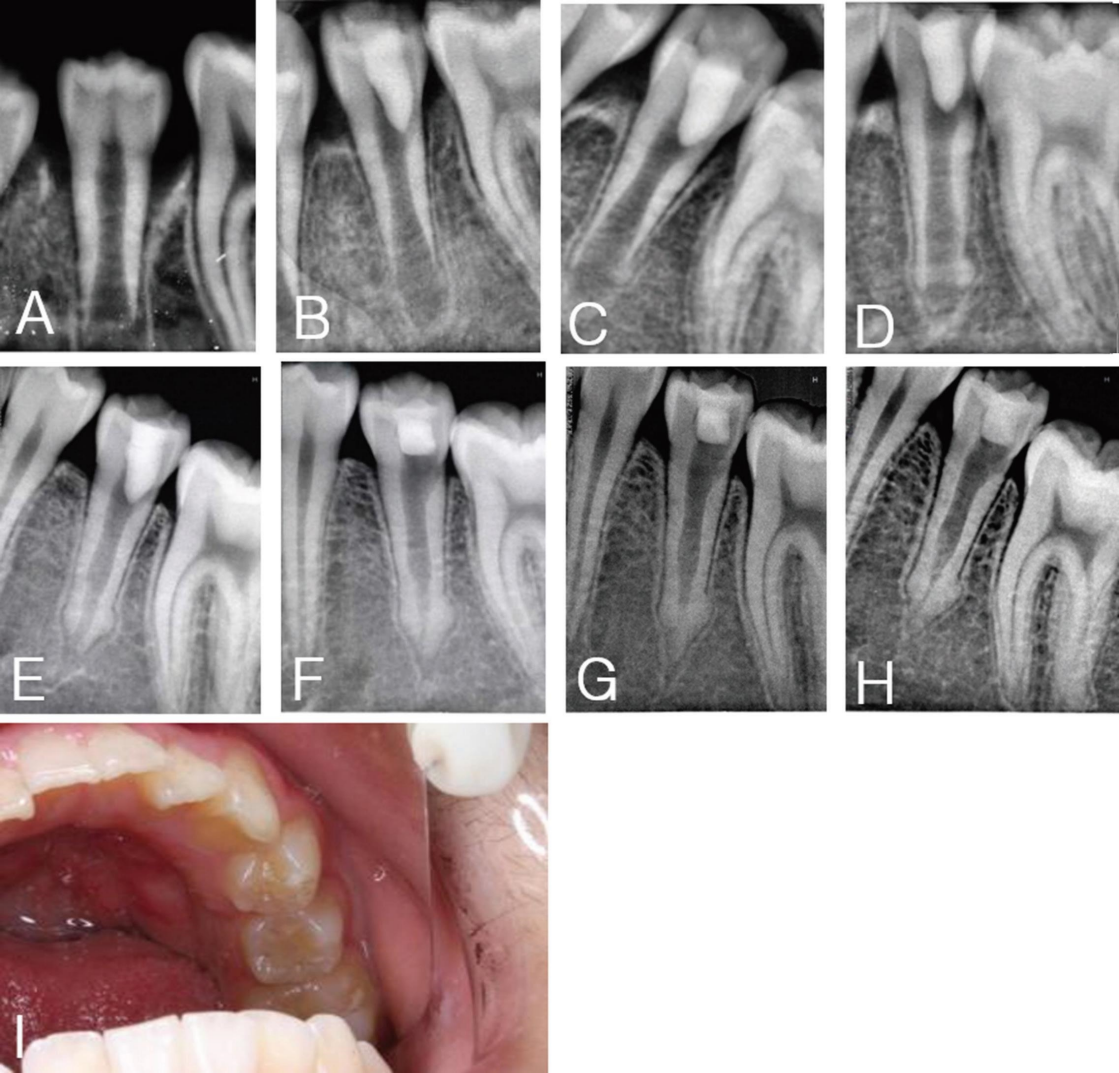
(A) Preoperative periapical radiograph; (B) 1‐month postoperative radiograph; (C) 3‐month postoperative radiograph; (D) 6‐month postoperative radiograph; (E) 2‐year postoperative radiograph; (F) 2.5‐year postoperative radiograph; (G) 3‐year postoperative radiograph; (H) 4‐year postoperative radiograph; and (I) intraoral photographs at the 4‐year follow‐up.

An 8‐year‐old male presented with a 4‐day history of pain in the left mandibular posterior region. Clinical examination revealed mild swelling in the left buccal area, normal mouth opening and mandibular movement, and a fractured dens evaginatus on the occlusal surface of Tooth 35. The tooth exhibited marked percussion sensitivity (++), Grade I mobility, delayed response to cold testing, and sensitivity to heat stimulation, accompanied by buccal gingival swelling.

Preoperative periapical radiographs demonstrated an immature root with an open apex corresponding to Nolla Stage 8, along with a periapical radiolucency and widening of the periodontal ligament space. A diagnosis of acute apical periodontitis associated with dens evaginatus was established.

Treatment was performed according to the standardized IVPT protocol described in Section 2.2. Briefly, following conservative access cavity preparation and removal of necrotic pulp tissue, the canal was irrigated with 1% NaOCl and sterile saline, DAP was placed over the remaining apical vital tissue, and the cavity was temporarily sealed with zinc oxide–eugenol cement.

At the 3‐month follow‐up, slight buccal gingival swelling persisted; however, the tooth was asymptomatic, with negative percussion findings, no mobility, and normal thermal responses. Radiographic examination showed reduction of the periapical radiolucency and narrowing of the apical foramen. The temporary restoration was removed, and the tooth was semipermanently restored with GIC.

At the 4‐year follow‐up, the tooth remained asymptomatic. Radiographs demonstrated continued root development with apical closure, dentin deposition in the apical region, and restoration of a normal periodontal ligament space, with no evidence of periapical radiolucency. Intraoral examination revealed normal tooth color and a well‐maintained restoration, indicating a successful outcome.

#### 2.4.2. Case 2 (Figure 2)

**Figure 2:**
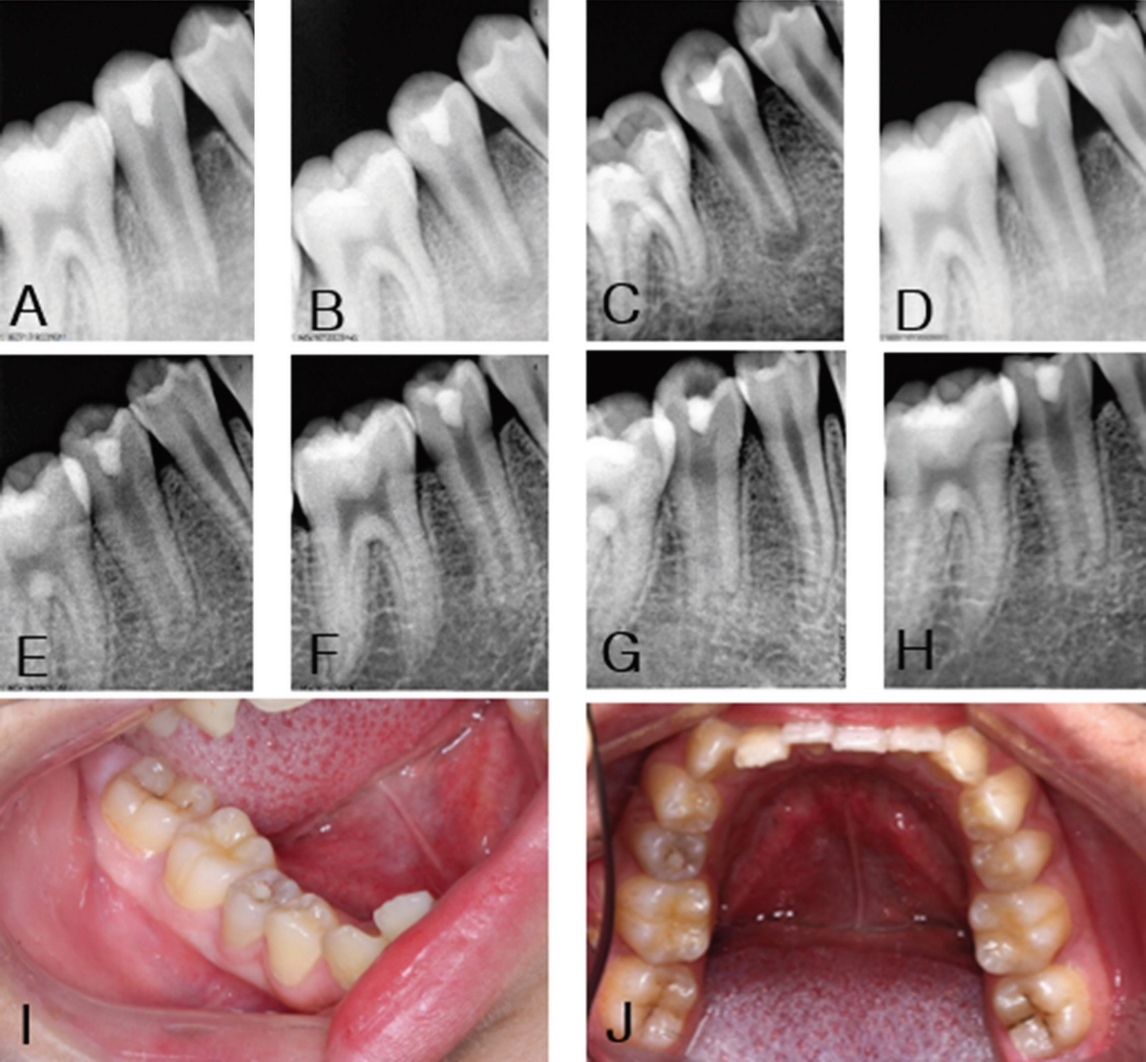
(A) First available radiograph obtained immediately after initial treatment; (B) 1‐month postoperative radiograph; (C) 3‐month postoperative radiograph; (D) 7‐month postoperative radiograph; (E) 10‐month postoperative radiograph; (F) 16‐month postoperative radiograph; (G) 19‐month postoperative radiograph; (H) 31‐month postoperative radiograph; and (I,J) intraoral photograph at the 31‐month follow‐up.

A 9‐year‐old male presented with a 4‐day history of pain in the right mandibular posterior region. Clinical examination revealed a dens evaginatus on the occlusal surface of Tooth 45, with marked percussion sensitivity (++), no mobility, and hypersensitivity to cold stimulation. The facial appearance was symmetrical, and mouth opening was normal.

It should be noted that a preoperative periapical radiograph could not be obtained because the patient had undergone emergency access opening at a local clinic prior to referral, which precluded standardized preoperative imaging. The first available radiograph after referral demonstrated an open apex corresponding to Nolla Stage 8, along with periapical radiolucency (Figure 2A).

Treatment was performed according to the standardized IVPT protocol described in Section 2.2, with no case‐specific modifications.

At the 3‐month follow‐up, the tooth was asymptomatic, with negative percussion findings and no mobility, and the temporary restoration remained intact. The temporary filling was subsequently removed, and the tooth was semipermanently restored with GIC.

At the 31‐month follow‐up, the tooth remained asymptomatic. Radiographic evaluation demonstrated continued root development with dentin deposition in the apical region and absence of periapical radiolucency. Intraoral examination showed normal tooth color and an intact restoration, indicating a successful outcome.

#### 2.4.3. Case 3 (Figure 3)

**Figure 3:**
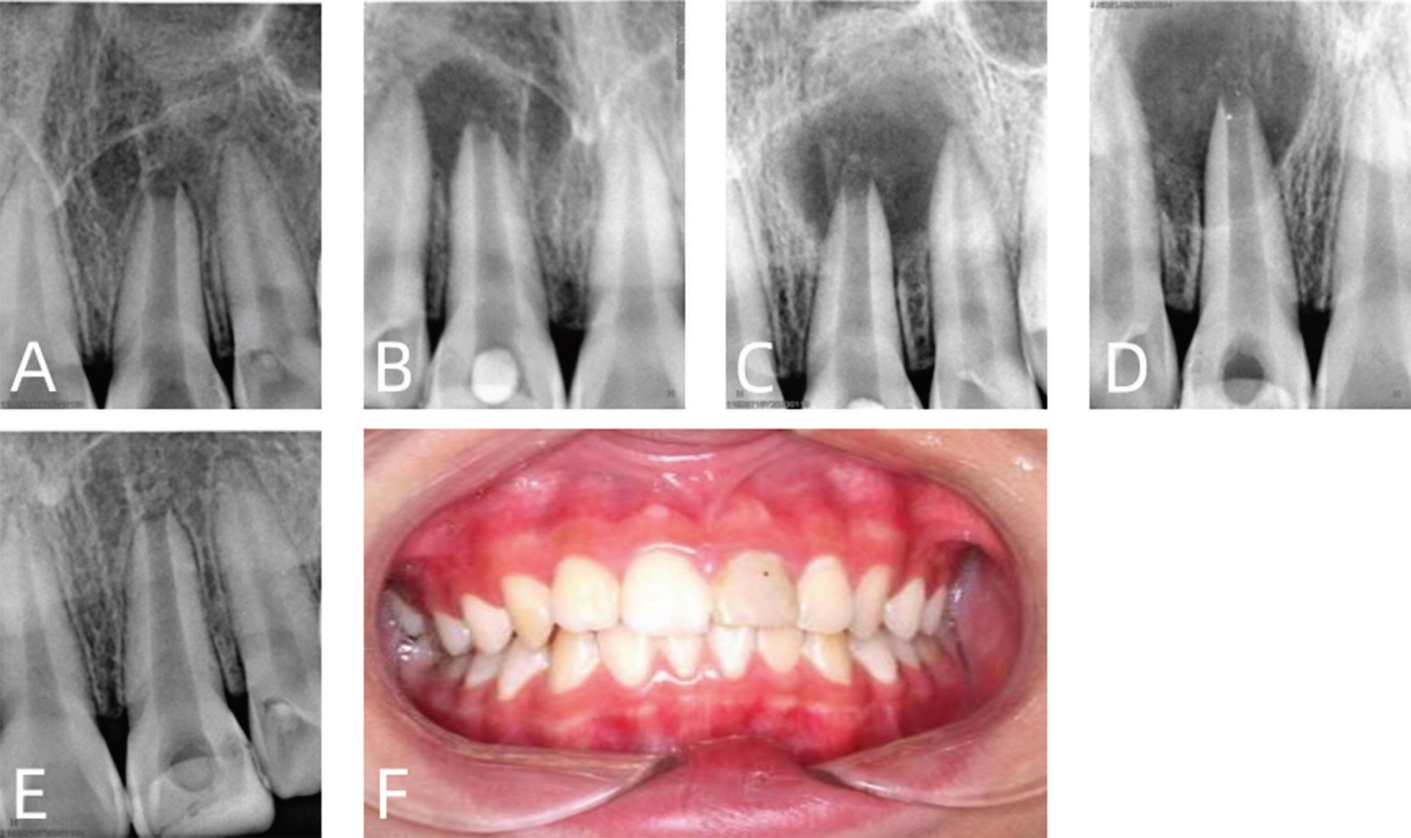
(A) Preoperative periapical radiograph; (B) second treatment visit; (C) 3‐month postoperative radiograph; (D) 12‐month postoperative radiograph; (E) 22‐month follow‐up periapical radiograph; and (F) intraoral photograph at the 22‐month follow‐up.

An 11‐year‐old male presented with several days of discomfort in the left maxillary anterior region. Clinical examination revealed mild upper lip swelling, normal mouth opening and mandibular movement, and noticeable oral malodor. Tooth 21 exhibited extensive carious involvement with marked probing tenderness (++), positive percussion sensitivity (+), positive palpation (++), gingival erythema, and Grade II mobility.

Preoperative periapical radiographs demonstrated a distinct periapical radiolucency associated with Tooth 21, with root development corresponding to Nolla Stage 9 and incomplete apical closure. A diagnosis of chronic apical periodontitis was established.

Treatment was performed according to the standardized IVPT protocol described in Section 2.2, with no case‐specific modifications.

At the 3‐month follow‐up, slight gingival erythema persisted, with equivocal percussion sensitivity, negative palpation findings, and Grade I mobility. The tooth was then semipermanently restored with GIC.

At the 12‐month follow‐up, mild coronal discoloration was noted on Tooth 21. The tooth remained asymptomatic with no signs of periapical pathology. The outcome was classified as partially successful.

#### 2.4.4. Case 4 (Figure 4)

**Figure 4:**
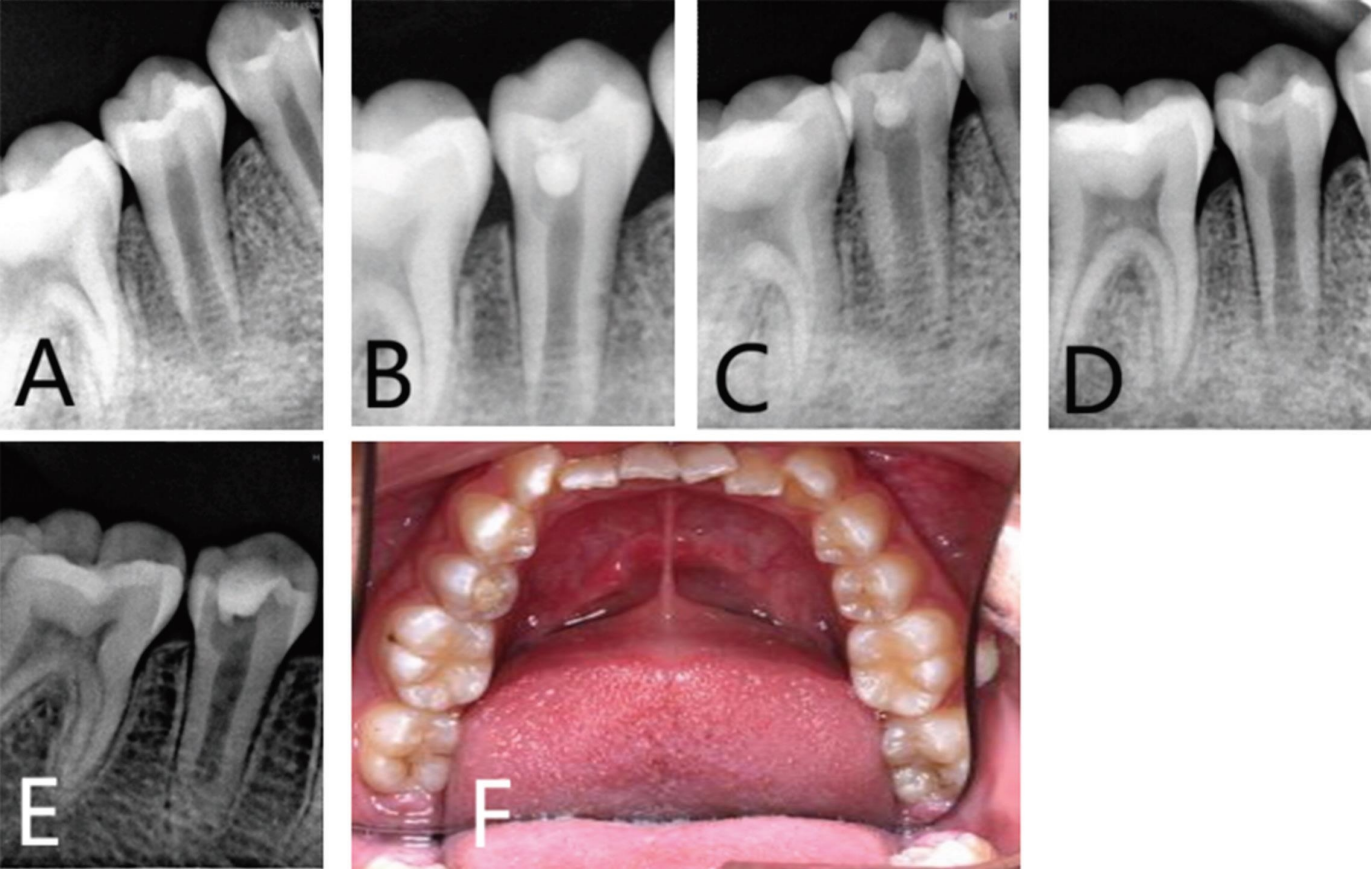
(A) Preoperative periapical radiograph; (B) 2‐month postoperative radiograph; (C) 9‐month postoperative radiograph; (D) 10‐month postoperative radiograph; (E) 27‐month follow‐up periapical radiograph; and (F) intraoral photograph at the 27‐month follow‐up.

A 12‐year‐old male presented with a 1‐week history of pain in the right mandibular posterior region. Clinical examination revealed mild swelling of the right buccal area with normal mouth opening. Intraoral examination showed a dens evaginatus on the occlusal surface of Tooth 45, with positive probing sensitivity, marked percussion pain (++), Grade I mobility, sensitivity to cold testing, and buccal gingival erythema.

Preoperative periapical radiographs demonstrated an immature root with an open apex corresponding to Nolla Stage 8. A diagnosis of acute apical periodontitis associated with dens evaginatus was established.

Treatment was performed according to the standardized IVPT protocol described in Section 2.2, with no case‐specific modifications.

At the 2‐month follow‐up, mild buccal gingival swelling and slight percussion tenderness persisted. The temporary restoration was removed, the canal was re‐evaluated, and the tooth was semipermanently restored with GIC.

At the 27‐month follow‐up, the tooth was asymptomatic, with normal color and texture, negative percussion and palpation responses, no mobility, and normal thermal responses. Radiographic examination demonstrated continued root development with apical dentin deposition and complete apical closure. The treatment outcome was considered successful.

## 3. Discussion

This study presents four cases of immature permanent teeth treated with IVPT, with a follow‐up period ranging from 22 to 48 months (mean: 32.5 months). Unlike RET, IVPT aims primarily to preserve residual vital apical tissues rather than regenerate a functional pulp‐like tissue through induced bleeding. All cases achieved resolution of clinical symptoms and periapical healing, whereas apical closure and continued root development were observed in three cases.

The favorable outcomes observed in this study may be attributed to the preservation and functional activity of SCAPs, which play a critical role in root development [12]. Previous studies have demonstrated that the apical papilla can remain viable even in the presence of pulp infection or necrosis. This biological resilience is supported by several factors, including collateral blood supply from surrounding periodontal tissues, lower metabolic demand compared with dental pulp, and close proximity to highly vascularized dental follicles, which facilitate nutrient and oxygen exchange [13].

Furthermore, Lin et al. [14] reported continued root maturation in immature teeth despite persistent periapical inflammation following failed RET, providing evidence that SCAPs can survive and function under compromised conditions. The findings of the present study are consistent with these observations, suggesting that effective infection control combined with preservation of apical vital tissues may enable continued root development in immature teeth.

In the present study, DAP was used instead of triple antibiotic paste (TAP), with the aim of achieving effective infection control while minimizing adverse effects. Although TAP, composed of ciprofloxacin, metronidazole, and minocycline, has been widely used due to its broad‐spectrum antimicrobial activity, the inclusion of minocycline has been associated with tooth discoloration through its interaction with calcium ions in dentin [15]. In contrast, DAP eliminates the risk of minocycline‐induced discoloration while maintaining satisfactory antimicrobial efficacy. Metronidazole is effective against obligate anaerobic bacteria commonly found in infected root canals, whereas ciprofloxacin provides coverage against aerobic and facultative anaerobic microorganisms. Notably, tooth discoloration occurred in Case 3 despite the use of DAP (metronidazole and ciprofloxacin) to avoid minocycline‐induced staining. Several factors may have contributed: (1) extensive carious involvement and pulpal necrosis prior to treatment may have caused intrinsic dentin staining; (2) residual blood products may have penetrated the dentinal tubules, as blood contamination has demonstrated high‐staining potential regardless of barrier materials [16]; and (3) zinc oxide–eugenol temporary cement has been shown to induce significant coronal discoloration, exhibiting the highest darkening effect among commonly used endodontic materials [17]. Therefore, although DAP eliminates the risk associated with minocycline‐induced discoloration, it cannot completely prevent discoloration caused by other intrinsic or treatment‐related factors.

Compared with conventional treatment strategies for immature permanent teeth, IVPT offers several potential advantages. In contrast to apexification, IVPT allows for a shorter treatment duration and avoids the structural weakening and canal calcification associated with prolonged calcium hydroxide application [18]. In addition, IVPT is technically less demanding than apical barrier techniques and does not require the placement of artificial apical barriers. Unlike conventional regenerative endodontic procedures [4, 19], IVPT does not intentionally induce intracanal bleeding as a scaffold for tissue regeneration, thereby simplifying the clinical procedure and improving its feasibility in primary care settings [20]. However, IVPT is not without limitations. Its success largely depends on appropriate case selection, particularly the presence of sufficient residual vital tissue and a favorable biological environment for stem cell survival [21]. Furthermore, effective infection control remains a critical determinant of treatment outcome [22]. In the present study, favorable clinical and radiographic outcomes were achieved when these conditions were met, suggesting that strict adherence to case selection criteria and infection control protocols is essential for the success of IVPT.

The findings of the present study indicate that IVPT may represent a simple and cost‐effective treatment option for immature permanent teeth with pulpal or periapical lesions, particularly for patients with limited socioeconomic resources or poor compliance with long‐term follow‐up. When effective infection control is achieved, the fundamental principle of this approach is to preserve residual vital tissue to the greatest extent possible, thereby harnessing the regenerative potential of immature teeth to support continued physiological root development [23, 24].

Current clinical practice guidelines issued by the American Association of Endodontists (AAE) and the European Society of Endodontology (ESE) recognize vital pulp therapy and regenerative endodontic procedures as viable treatment alternatives for immature permanent teeth with pulp necrosis. According to the AAE guidelines, the success of regenerative procedures is measured by three levels of goal: (1) primary goal: elimination of inflammatory symptoms and evidence of bone healing; (2) secondary goal: increased root length and wall thickness; and (3) tertiary goal: positive response to dental pulp testing [25]. Similarly, the ESE position statement on revitalization procedures (2016) defines successful outcomes as including (1) absence of inflammatory symptoms, (2) radiographic evidence of bone healing, (3) increased root length and thickness, (4) absence of external root resorption, (5) positive response to dental pulp test, (6) signs of new periodontal ligament formation, and (7) unchanged crown color [26]. In the present case series, all four cases achieved the primary goal (elimination of symptoms and periapical healing), and three cases demonstrated radiographic evidence consistent with the secondary goal of continued root maturation. The IVPT protocol described herein aligns with these guideline recommendations, particularly regarding the preservation of apical vital tissues and the use of a minocycline‐free antibiotic regimen. Nevertheless, standardized protocols for IVPT, including optimal medication concentration, dressing duration, and definitive restoration timing, have yet to be established through rigorous prospective clinical trials. Future studies integrating these guidelines with long‐term clinical data are warranted to refine treatment protocols and establish evidence‐based recommendations for IVPT in immature permanent teeth.

Several limitations of this case series should be acknowledged. First, all four cases involved male patients, which was coincidental and not by design; the absence of female patients limits the generalizability of the findings, and future studies should include a more balanced gender distribution to evaluate potential sex‐related differences in treatment outcomes. Second, GIC was used as the definitive coronal restoration in all cases. Although GIC provides adequate sealing, fluoride release, and chemical adhesion, it offers lower mechanical strength and wear resistance compared with composite resin or giomer materials. In this case series, GIC was selected as a transitional restoration in growing patients, with the understanding that definitive composite restorations may be considered after complete root maturation and when patient cooperation allows.

## 4. Conclusions

This case series demonstrates that IVPT using DAP can achieve favorable clinical and radiographic outcomes in immature permanent teeth with pulpal or periapical pathology, including periapical healing and continued root development in most cases during long‐term follow‐up. The use of DAP provides effective infection control while reducing the risk of tooth discoloration. However, further large‐scale prospective studies are required to confirm its long‐term efficacy and safety.

**Nomenclature**
DAP: double antibiotic paste
IVPT: inflammatory vital pulp therapy
MTA: mineral trioxide aggregate
NaOCl: sodium hypochlorite
RET: regenerative endodontic treatment
SCAPs: stem cells from the apical papilla
TAP: triple antibiotic paste

## Author Contributions

Y.W. collected the clinical data and drafted the manuscript. W.Z. participated in the clinical treatments and radiographic imaging. X.Z. designed the study and critically revised the manuscript.

## Funding

No funding was received for this manuscript.

## Disclosure

All authors read and approved the final manuscript.

## Ethics Statement

The study protocol was approved by the Science and Technology Ethics Committee of the Affiliated Hospital of Yanbian University (Approval No.: 20260261) and conducted in accordance with the Declaration of Helsinki. Written informed consent for treatment and publication was obtained from the parents or legal guardians of all patients. All patient data were anonymized prior to analysis, and no identifiable patient information or facial photographs are included in this article.

## Consent

Written informed consent for publication of identifying images or other personal or clinical details was obtained from parents/legal guardians of the patients.

## Conflicts of Interest

The authors declare no conflicts of interest.

## Data Availability

The datasets used and/or analyzed during the current study are available from the corresponding author on reasonable request.

## References

[1] Burns L. E. , Gencerliler N. , Terlizzi K. , Solis-Roman C. , Sigurdsson A. , and Gold H. T. , Apexification Outcomes in the United States: A Retrospective Cohort Study, Journal of Endodontics. (2023) 49, no. 10, 1269–1275, 10.1016/j.joen.2023.07.020, 37517583. 37517583 PMC10543604

[2] Murray P. E. , Review of Guidance for the Selection of Regenerative Endodontics, Apexification, or Apicoectomy for Treatment of Immature Permanent Teeth, Journal of the American Dental Association. (2020) 151, no. 6, 416–422, 10.1111/iej.13809.32450980

[3] Andreasen J. O. , Farik B. , and Munksgaard E. C. , Long-Term Calcium Hydroxide as a Root Canal Dressing May Increase Risk of Root Fracture, Dental Traumatology. (2002) 18, no. 3, 134–137, 10.1034/j.1600-9657.2002.00097.x, 12110105. 12110105

[4] Lin L. M. and Kahler B. , A Review of Regenerative Endodontics: Current Protocols and Future Directions, Journal of Istanbul University Faculty of Dentistry. (2017) 51, S41–S51, 10.17096/jiufd.53911, 29354308. 29354308 PMC5750827

[5] Schmalz G. , Widbiller M. , and Galler K. M. , Clinical Perspectives of Pulp Regeneration, Journal of Endodontics. (2020) 46, no. 9, S161–S174, 10.1016/j.joen.2020.06.037. 32950188

[6] Xiao W. , Shi W. T. , and Wang J. , Study of Vital Inflamed Pulp Therapy in Immature Permanent Teeth With Irreversible Pulpitis and Apical Periodontitis, Zhonghua Kou Qiang Yi Xue Za Zhi. (2022) 57, no. 3, 287–291, 10.3760/cma.j.cn112144-20211223-00563, 35280007. 35280007

[7] Kyaw M. S. , Kamano Y. , Yahata Y. , Tanaka T. , Sato N. , Toyama F. , Noguchi T. , Saito M. , Nakano M. , Harada F. , and Saito M. , Endodontic Regeneration Therapy: Current Strategies and Tissue Engineering Solutions, Cells. (2025) 14, no. 6, 10.3390/cells14060422, 40136671. PMC11941292 40136671

[8] Martin D. E. , De Almeida J. F. , Henry M. A. et al., Concentration-Dependent Effect of Sodium Hypochlorite on Stem Cells of Apical Papilla Survival and Differentiation, Journal of Endodontics. (2014) 40, no. 1, 51–55, 10.1016/j.joen.2013.07.026, 24331991. 24331991

[9] Ulin C. , Magunacelaya-Barria M. , Dahlén G. , and Kvist T. , Immediate Clinical and Microbiological Evaluation of the Effectiveness of 0.5% Versus 3% Sodium Hypochlorite in Root Canal Treatment: A Quasi-Randomized Controlled Trial, International Endodontic Journal. (2020) 53, no. 5, 591–603, 10.1111/iej.13258, 31808947. 31808947

[10] Khoroushi M. and Keshani F. , A Review of Glass-Ionomers: From Conventional Glass-Ionomer to Bioactive Glass-Ionomer, Dental Research Journal. (2013) 10, no. 4, 411–420, 24130573. 24130573 PMC3793401

[11] Bukhari S. , Kohli M. R. , Setzer F. , and Karabucak B. , Outcome of Revascularization Procedure: A Retrospective Case Series, Journal of Endodontics. (2016) 42, no. 12, 1752–1759, 10.1016/j.joen.2016.06.021. 27726882

[12] Kang J. , Fan W. , Deng Q. , He H. , and Huang F. , Stem Cells From the Apical Papilla: A Promising Source for Stem Cell-Based Therapy, BioMed Research International. (2019) 2019, 6104738, 10.1155/2019/6104738. 30834270 PMC6374798

[13] Diogenes A. and Hargreaves K. M. , Microbial Modulation of Stem Cells and Future Directions in Regenerative Endodontics, Journal of Endodontics. (2017) 43, no. 9, S95–S101, 10.1016/j.joen.2017.07.012, 28844309. 28844309

[14] Lin L. M. , Kim S. G. , Martin G. , and Kahler B. , Continued Root Maturation Despite Persistent Apical Periodontitis of Immature Permanent Teeth After Failed Regenerative Endodontic Therapy, Australian Endodontic Journal. (2018) 44, no. 3, 292–299, 10.1111/aej.12252, 29336522. 29336522

[15] Kim J. H. , Kim Y. , Shin S. J. , Park J. W. , and Jung I. Y. , Tooth Discoloration of Immature Permanent Incisor Associated With Triple Antibiotic Therapy: A Case Report, Journal of Endodontics. (2010) 36, no. 6, 1086–1091, 10.1016/j.joen.2010.03.031, 20478471. 20478471

[16] Fagogeni I. , Metlerska J. , Lipski M. , Falgowski T. , Maciej G. , and Nowicka A. , Materials Used in Regenerative Endodontic Procedures and Their Impact on Tooth Discoloration, Journal of Oral Science. (2019) 61, no. 3, 379–385, 10.2334/josnusd.18-0467, 31378754. 31378754

[17] Xavier S. R. , Pilownic K. J. , Gastmann A. H. , Echeverria M. S. , Romano A. R. , and Geraldo P. F. , Bovine Tooth Discoloration Induced by Endodontic Filling Materials for Primary Teeth, International Journal of Dentistry. (2017) 2017, 7401962, 10.1155/2017/7401962. 28479918 PMC5396471

[18] Wikström A. , Brundin M. , Lopes M. F. , El Sayed M. , and Tsilingaridis G. , What Is the Best Long-Term Treatment Modality for Immature Permanent Teeth With Pulp Necrosis and Apical Periodontitis?, European Archives of Paediatric Dentistry. (2021) 22, no. 3, 311–340, 10.1007/s40368-020-00575-1, 33420674. 33420674 PMC8213569

[19] Aksoy D. and Kocak S. , Management Strategies for Teeth With Open Apex: From Apexification to Regenerative Endodontics, Cumhuriyet Dental Journal. (2025) 28, no. 3, 462–468, 10.7126/cumudj.1728795.

[20] Wei X. , Yang M. , Yue L. , Huang D. , Zhou X. , Wang X. , Zhang Q. , Qiu L. , Huang Z. , Wang H. , Meng L. , Li H. , Chen W. , Zou X. , and Ling J. , Expert Consensus on Regenerative Endodontic Procedures, International Journal of Oral Science. (2022) 14, no. 1, 10.1038/s41368-022-00206-z, 36450715. PMC9712432 36450715

[21] Murray P. E. , Review of Guidance for the Selection of Regenerative Endodontics, Apexogenesis, Apexification, Pulpotomy, and Other Endodontic Treatments for Immature Permanent Teeth, International Endodontic Journal. (2023) 56, no. S2, 188–199, 10.1111/iej.13809, 35929348. 35929348

[22] Siqueira J. F. , Aetiology of Root Canal Treatment Failure: Why Well-Treated Teeth Can Fail, International Endodontic Journal. (2001) 34, no. 1, 1–10, 10.1046/j.1365-2591.2001.00396.x, 11307374. 11307374

[23] Sonoyama W. , Liu Y. , Yamaza T. , Tuan R. S. , Wang S. , Shi S. , and Huang G. T. J. , Characterization of the Apical Papilla and Its Residing Stem Cells From Human Immature Permanent Teeth: A Pilot Study, Journal of Endodontics. (2008) 34, no. 2, 166–171, 10.1016/j.joen.2007.11.021, 18215674. 18215674 PMC2714367

[24] Cui D. , Yu S. , Zhou X. , Liu Y. , Gan L. , Pan Y. , Zheng L. , and Wan M. , Roles of Dental Mesenchymal Stem Cells in the Management of Immature Necrotic Permanent Teeth, Frontiers in Cell and Developmental Biology. (2021) 9, 666186, 10.3389/fcell.2021.666186, 34095133. 34095133 PMC8170050

[25] American Association of Endodontists , AAE Clinical Considerations for a Regenerative Procedure, 2021, https://www.aae.org/specialty/clinical-resources/regenerative-endodontics.

[26] Galler K. M. , Krastl G. , Simon S. , Van Gorp G. , Meschi N. , Vahedi B. , and Lambrechts P. , European Society of Endodontology Position Statement: Revitalization Procedures, International Endodontic Journal. (2016) 49, no. 8, 717–723, 10.1111/iej.12629, 26990236. 26990236

